# Transrectal prostate biopsy complications: a prospective single center study in a mid-income country

**DOI:** 10.31744/einstein_journal/2025AO1437

**Published:** 2025-08-08

**Authors:** Allan Jefferson Schollemberg, Flavio Lobo Heldwein, Suelen dos Santos, Vítor Maurício Merlin Maschietto, Erick Schnorrenberger, Kleber Reinert, Gabriela Garcia Korczaguin, Marcelo Langer Wroclawski

**Affiliations:** 1 Hospital Governador Celso Ramos Florianópolis SC Brazil Hospital Governador Celso Ramos, Florianópolis, SC, Brazil.; 2 Department of Urology Universidade Federal de Santa Catarina Florianópolis SC Brazil Department of Urology, Universidade Federal de Santa Catarina, Florianópolis, SC, Brazil.; 3 Universidade do Sul de Santa Catarina Palhoça SC Brazil Universidade do Sul de Santa Catarina, Palhoça, SC, Brazil.; 4 Hospital Israelita Albert Einstein São Paulo SP Brazil Hospital Israelita Albert Einstein, São Paulo, SP, Brazil.; 5 Department of Urologic Oncology A Beneficência Portuguesa de São Paulo São Paulo SP Brazil Department of Urologic Oncology, A Beneficência Portuguesa de São Paulo, São Paulo, SP, Brazil.

**Keywords:** Urinary tract infections, Image-guided biopsy, Drug resistance, Mass screening, Prostatic diseases, Early detection of cancer, Risk factors

## Abstract

**Objective:**

To identify complications following transrectal posterior biopsies in a public Brazilian reference center, and to identify the risk factors associated with complications.

**Methods:**

This is a prospective cohort study that employed a form designed by the Global Prevalence Infections in Urology study. Data from 1,043 consecutive patients who underwent transrectal prostate biopsy at a single Brazilian center were analyzed, including patient characteristics, procedural characteristics, and self-assessed complications at 28 days. Complications were categorized according to the Clavien-Dindo (CD) classification.

**Results:**

Most bleeding complications were mild (CD Grade 1), although 1.5% reported Grades 2–3. Hypertension, younger age, and anticoagulant use were associated with bleeding (all p<0.001). A total of 4.7% reported infections (CD Grade 2: 0.5%, CD Grade 3: 3.6%, and CD Grade 4 [sepsis]: 0.6%), with risk factors being indwelling catheter use, recent urinary tract infection (p<0.001 for both), and quinolone use (OR= 3.01, 95%CI= 1.15–7.80, p=0.03). Urinary retention was observed in 4.1% (Grade 2), with severe symptoms (p=0.009), prostates >89mL (p=0.001), and prostatic protrusion ≥10mm (p=0.001) being associated with it.

**Conclusion:**

Clinically significant (CD Grades 3–5) adverse effects of transrectal prostate biopsy are rare. Careful pre-procedure evaluation of antimicrobial use, particularly quinolones, along with the assessment of identified risk factors is essential for counseling patients and reducing potential risks.

## Highlights

■ This prospective cohort study assessed prostate biopsy complications in 1,043 men.

■ The major outcomes included bleeding (Clavien-Dindo Grades 2–3 = 1.5%), infections (4.7%), and urinary retention (4.1%).

■ The key risk factors for infection were recent quinolone use, indwelling catheters, and high PSA levels.

■ Acute urinary retention was linked to untreated lower urinary tract symptoms, prostate volume, intravesical prostatic protrusion, and bladder wall thickness.

## In Brief

This prospective study conducted in a mid-income country evaluated the risk factors associated with complications after transrectal prostate biopsy and highlighted significant correlations with hemorrhagic events, infections, and acute urinary retention.

## Graphical abstract

**Figure:**
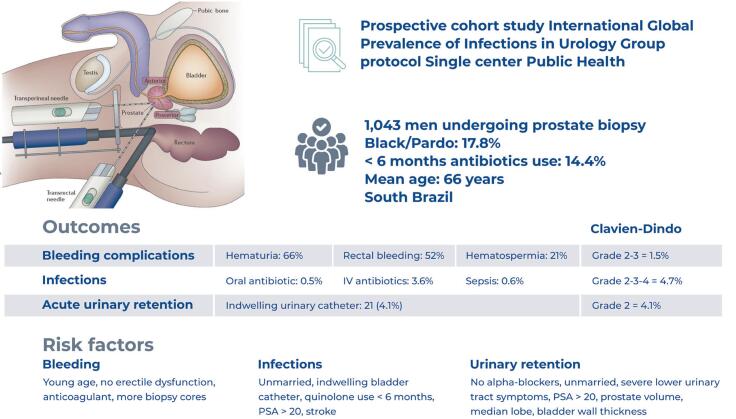

## INTRODUCTION

Prostate cancer (PCa) is once again on the rise, following controversial recommendations from the U.S. Preventive Services Task Force.^(1,2)^ In the USA, the incidence is about 299,000 new cases annually, with around 35,200 deaths each year.^(1)^ Globally, PCa is the fifth most deadly cancer among men. In Brazil, data from the National Cancer Institute indicate a prevalence of 52 cases per 100,000 men, with this number increasing to more than 90 per 100,000 men in the southern region, where life expectancy is the highest nationwide.^(3)^

Currently, ultrasound-guided prostate biopsy (PBx) is considered the gold standard for the diagnosis of prostate cancer. Indeed, the current European Association of Urology guidelines recommend moving from the transrectal approach to the transperineal approach; they also recommend that biopsy samples target lesions and perilesional areas, guided by magnetic resonance imaging (MRI) findings.^(4)^Initiatives such as Trexit also advocate abandoning the transrectal approach in favor of the transperineal route.

After PBx, many patients develop minor (self-limiting) complications, with hematuria and rectal bleeding being the most common. According to the literature, up to 5% of patients experience major complications that require intervention and/or hospitalization.^(5)^

One of the most concerning complications are urinary tract infections, the incidence of which has increased in recent years.^(5)^Increased bacterial resistance in urology departments is a permanent concern. Further, the use of quinolones, a common treatment for urinary tract infections, has been investigated in Europe, and current guidelines recommend against its use in treating uncomplicated urinary tract infections.^(4)^

### OBJECTIVE

To identify complications following transrectal prostate biopsies in a public Brazilian reference center, the risk factors associated with complications were identified.

## METHODS

This single-center, prospective, observational cohort study was conducted at the *Centro de Pesquisas Oncologicas* (Oncology Research Center) of the Santa Catarina Department of Health in Florianopolis, Santa Catarina, Brazil. The study population consisted of patients undergoing transrectal PBx with periprostatic nerve block (lidocaine 1%) plus topical intrarectal anesthesia with 10cc 2% lidocaine gel. Antibiotic prophylaxis was administered using a 3-day oral regimen of levofloxacin (500mg once per day), which was initiated 4h prior to the procedure. Rectal cultures were obtained from patients who reported the recent use of quinolones to screen for fluoroquinolone-resistant organisms, and their prophylaxis was adjusted according to the results. All patients underwent rectal preparation with 130mL fleet-enema 1 hour prior. Twelve core radiomic (with or without target samples) biopsies were performed by a single experienced urologist using an 18G coaxial disposable needle with a penetration depth of 18–22mm, mounted in a reusable biopsy gun.

The inclusion criteria were male sex with a clinical recommendation for prostate biopsy, signed informed consent, and fit to undergo transrectal biopsy under local anesthesia. The exclusion criteria was that patients who did not consent to or tolerate the procedure under local anesthesia were excluded. This study recruited patients with elevated prostate-specific antigen (PSA) levels, with or without prior multiparametric MRI and/or suspicious findings on digital rectal examination of the prostate.

Although data collection took place between April 2009 and January 2020, only patients enrolled between April 2013 and April 2016 were included in this analysis, as later records were gathered but not fully digitized, thereby preventing their inclusion. The patients were interviewed during routine biopsy screening consultations. Data were collected after obtaining informed consent, and all procedures were conducted during regular outpatient consultations. A standardized data collection questionnaire (Global Prevalence of Infections in Urology protocol)^(6)^ was used, which included variables related to demographic characteristics, prebiopsy medical history, biopsy technique, and follow-up information. Because most of these patients were referrals, the 28-day follow-up data were self-reported by the participants. Complications were categorized according to the Clavien-Dindo complication scale.

The results were presented as numbers and percentages for binary and categorical variables, mean (standard deviation) for normally distributed continuous variables, and median (interquartile range) for non-normally distributed continuous variables. Data were analyzed using appropriate statistical tests (Pearson’s chi-squared test, Student’s *t*-test, or Mann-Whitney U test) performed using SPSS version 29.0 (SPSS Inc., 2024). Statistical significance was set at p<0.05.

Ethical approval was obtained from the Local Ethics Committee of *Centro de Pesquisas Oncológicas* (CAAE: 10825513.8.0000.5355; #3.377.279), and informed consent was obtained from all participants. The authors declare no conflicts of interest.

## RESULTS

Of the 1,043 patients evaluated, 101 (11.5%) were illiterate, and 508 (57.7%) had completed primary school or less. Regarding ethnicity, 156 (17.8%) self-identified as black or of mixed race. Thirty-five (4.1%) patients had a body mass index (BMI) >35. Notably, the 66 patients reporting recent antibiotic use represent 8.1% of the total cohort (n=805) and 57% of the 116 respondents with valid data for this variable. Regarding PSA levels, 89/752 (11.8%) had values above 20ng/dL. Finally, 34/708 (4.8%) underwent biopsy based solely on abnormal digital rectal examination findings. Demographic details and biopsy indications are listed in table 1.

**Table 1:** Socio-demographic characteristics of patients undergoing prostate biopsy

Patients (n)	1,043
Age mean (SD)	66 (7.7)
Education level, n (%)	
Illiterate	101 (11.5)
Primary education or less	508 (57.7)
Secondary education or less	195 (21.1)
High School or equivalent	46 (5.2)
Some university education	7 (0.8)
University degree	24 (2.7)
Marital status, n (%)	
Married	704 (75.5)
Divorced	97 (11)
Single	33 (3.7)
Widowed	50 (5.7)
Ethnicity, n (%)	
White	718 (82)
Black/pardo (multiracial)	156 (17.8)
Asian	2 (0.2)
BMI, n (%)	
Underweight (BMI less than 20)	40 (4.6)
Normal weight (BMI 21–25)	272 (31.6)
Overweight (BMI 26–30)	368 (42.7)
Obesity Class I (BMI 30–35)	146 (17)
Obesity Class II or higher (BMI >35)	35 (4.1)
Family history of prostate cancer, n (%)	149 (16.9)
Family history of other cancers	83 (25.6)
Recent antibiotic use, n (%)	116 (14.4)
Recent use of quinolone, n (%)	66 (8.1)
Recent urinary tract infections, n (%)	34 (4.2)
Urinary catheter, n (%)	27 (3.4)
Median PSA (p25–p75)	8.0 (5.5–12.9)
PSA (ng/dL)	
<3	41 (5.5)
3–10	429 (57)
10–20	193 (25.7)
>20	89 (11.8)
Suspicious rectal examination, n (%)	364 (34.9)
Indication	
PSA only	347 (48.9)
DRE only	34 (4.8)
Abnormal PSA + DRE	330 (46)
Prior multiparametric MRI 1.5T	160 (14.7)

SD: standard deviation; BMI: body mass index; PSA: prostate-specific antigen; MRI: magnetic resonance imaging; DRE: digital rectal examination.

Overall, adverse events (AE) were common. Bleeding events were the most frequently reported. In 64.6% of the patients reporting hematuria, 52.1% experienced rectal bleeding and 21% experienced hemospermia, most of which were classified as Clavien-Dindo Grade 1. Eight (1.5%) patients reported Grade 2–3 AE due to hematuria requiring catheterization with bladder irrigation and one requiring blood transfusion. Further analysis revealed significant associations between hemorrhagic complications and younger age (p<0.001), hypertension (p<0.001), and anticoagulant use (p<0.001). In univariate analyses, anticoagulant use was significantly associated with Grade >1 bleeding (odds ratio [OR] = 8.552, 95% confidence interval [95% CI]: 2.056–35.566, p<0.001), as was the diagnosis of hypertension (OR= 2.708, 95%CI= 1.659–4.421, p<0.001). Moreover, patients with PSA levels >20ng/dL had a higher likelihood of hemorrhagic complications (p=0.002), whereas those with <12 biopsy fragments showed a reduced risk (p<0.001) (Table 2).

**Table 2:** Clinicopathological characteristics stratified by vascular complications

	Vascular complications		Total	p value
	No	Yes		
Number of patients	109 (20.3)	428 (79.7)	537	-
Mean age (SD)	68.5 (7.7)	65.0 (7.4)	65.8 (7.7)	<0.001
Age in years, n (%)				0.001
<60	15 (13.8)	118 (27.6)	133 (24.8)	
60–70	47 (43.1)	192 (44.9)	239 (44.5)	
>70	47 (43.1)	118 (27.6)	165 (30.7)	
Hypertension, n (%)	24 (21.6)	186 (42.8)	210 (38.5)	<0.001
*Diabetes mellitus*, n (%)	13 (11.7)	74 (17)	87 (15.9)	0.17
IPSS (%)				0.08
Normal	11 (20.8)	34 (10.2)	45 (11.7)	
Mild (1-7)	14 (26.4)	103 (31)	117 (30.4)	
Moderate (8-19)	14 (26.4)	124 (37.3)	138 (35.8)	
Severe (20-35)	14 (26.4)	71 (21.4)	85 (22.1)	
Normal IIEF5, n (%)	4 (9.5)	62 (19.6)	66 (18.4)	0.008
Anticoagulant, n (%)	2 (1.8)	59 (13.6)	61 (11.2)	<0.001
Statin, n (%)	4 (13.3)	36 (27.7)	40 (25)	0.10
PSA (%) (ng/mL)				0.02
<3	5 (4.7)	17 (4.1)	22 (4.2)	
3–10	49 (45.8)	265 (63.5)	314 (59.9)	
10–20	31 (29)	98 (23.5)	128 (24.6)	
>20	22 (20.6)	37 (8.9)	59 (11.3)	
Median prostate volume (mL) (p25–p75)	51 (35–89)	49 (35–71)	51 (36–77)	0.60
Number of fragments – mean (SD)	11 (4.3)	13.7 (3.1)	13.5 (3.4)	<0.001
<12 fragments in the biopsy,n (%)	20 (37.3)	26 (10.3)	46 (15.1)	<0.001

SD: standard deviation; PSA: prostate-specific antigen; IPSS: International Prostate Symptoms Score; IIEF5: International Index of Erectile Function 5.

Post-biopsy infections were observed in 27 patients (5%), most of which were classified as major (Grade 3–4 AE). Four patients developed urosepsis (Grade 4) and 20 were hospitalized for intravenous antibiotic therapy. Significant risk factors for infectious complications included bladder catheterization (p<0.001), history of urinary tract infections (p<0.001), and quinolone use 6 months before PBx (p=0.01). In a univariate risk analysis, the odds ratio for infection in patients who used quinolones in the preceding 6 months was 3.01 (95%CI= 1.15–7.80, p=0.03). In contrast, being married was associated with a lower rate of infection (p<0.001), while elevated PSA levels above 20ng/dL were also correlated with an increased infection risk (p=0.002) (Table 3).

**Table 3:** Clinicopathological characteristics stratified by infectious complications

	Infectious complications		Total	p value
	No	Yes		
Number of patients	530 (95)	28 (5)	558	
Mean age (SD)	65.7 (7.6)	67.2 (8.1)	66 (7.6)	0.33
>70 years (valid %)	153 (30.1)	11 (42.3)	164 (30.7)	0.40
Marital status; married, n (%)	375 (80.5)	17 (6.8)	392 (79.8)	<0.001
Ethnicity				0.21
White	378 (94.7)	22 (5,3)	399 (82.3)	
Black/ pardo (multiracial)	83 (97.3)	2 (2.7)	85 (17.5)	
Bladder catheterization (last 6 months)	18 (3.4)	6 (21.4)	24 (4.3)	<0.001
Antibiotic therapy (last 6 months)	78 (14.7)	6 (21.4)	84 (15.1)	0.33
Quinolone (last 6 months)	43 (8.3)	6 (21.4)	49 (9)	0.01
Urinary tract infections (last 6 months)	22 (4.2)	5 (17.9)	27 (4.8)	<0.001
Normal IIEF-5	121 (35.6)	3 (18.8)	124 (34.8)	0.06
PSA (%) (ng/mL)				0.002
<3	21 (4.2)	1 (3.7)	22 (4.2)	
3–10	306 (61.8)	8 (29.6)	314 (60.2)	
10–20	118 (23.8)	12 (37)	12 (24.5)	
>20	56 (10.1)	8 (29.6)	58 (11.1)	
Median prostate volume in mL (p25–p75)	48 (35–70)	62 (32–98)	51 (36–73)	0.12
Number of fragments – mean (SD)	13.4 (3.3)	12.6 (4.4)	13.5 (3.4)	0.22
*Diabetes mellitus*, n (%)	85 (16.5)	1 (3.6)	86 (15.8)	0.07
Stroke, n (%)	15 (2.9)	3 (10.7)	18 (3.3)	0.02

SD: standard deviation; IIEF5: International Index of Erectile Function 5.

**Figure 1:**
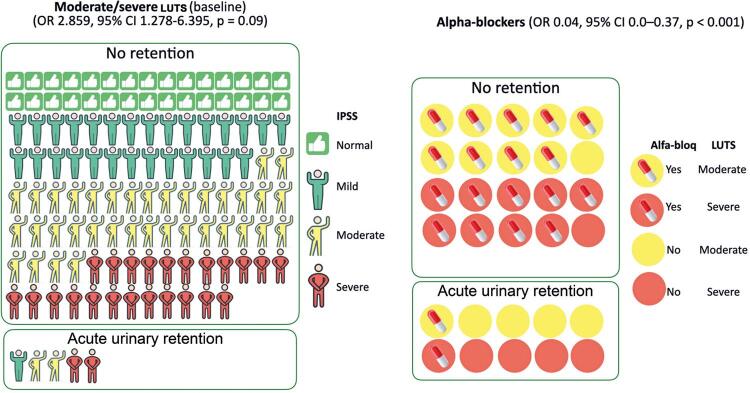
International Prostate Symptoms Score at baseline (left) and current use of alpha-blockers (right) and their correlation with acute urinary retention LUTS: lower urinary tract symptoms.

Acute urinary retention (AUR) was observed in 22 patients (4.1%). All patients required catheterization (Grade 2 AE). Severe lower urinary tract symptoms (LUTS) were significantly associated with the occurrence of AUR (p=0.002). Prostate volume greater than 89mL (p=0.001) was identified as a significant risk factor for AUR. In univariate analyses, urinary retention was associated with the presence of an intravesical prostatic protrusion (IPP) greater than 10mm (OR= 3.883, 95%CI= 1.285–11.731, p=0.02), bladder wall thickening ≥3mm (OR= 9.506, 95%CI= 1.147–78.790, p=0.02), and moderate to severe preexisting LUTS (OR= 2.859, 95%CI= 1.278–6.395, p=0.09). Conversely, the current use of alpha-blockers was a protective factor against the development of urinary retention (OR= 0.04, 95%CI= 0.0–0.37, p<0.001) (Table 4 and Figure 1).

**Table 4:** Clinicopathological characteristics stratified by acute urinary retention

	Acute urinary retention		Total	p value
	No	Yes		
Number of patients	488 (95.9)	22 (4.1)	510	
>70 years, n (%)	150 (30.7)	5 (23.8)	155 (30.5)	0.70
Alpha-blocker use, n (%)	67 (14.8)	1 (4.5)	68 (13.8)	<0.001
Inhabitant city >60km, n (%)	102 (95.3)	5 (4.7)	107 (23.3)	0.94
Marital status; married (%)	358 (80.4)	14 (63.6)	372 (79.7)	0.01
Severe LUTS (IPSS 20–35)	69 (19.7)	6 (40)	75 (20.5)	0.02
PSA (ng/mL), n (%)				0.02
<3	20 (4.2)	1 (4.5)	21 (4.2)	
3–10	300 (62.9)	9 (40.5)	309 (61.9)	
10–20	117 (24.5)	7 (31.8)	124 (24.8)	
>20	40 (8.4)	5 (22.7)	45 (9)	
Hematuria after ≥ 3 days, n (%)	129 (25.9)	9 (40.9)	138 (26.5)	0.11
Median prostate volume in mL (p25–p75)	47 (35–69)	89 (59–109)	51 (36–73)	0.01
Median lobe, n (%)	45 (16.2)	6 (42.9)	51 (17.5)	0.02
Bladder wall thickening ≥3mm, n (%)	81 (42.4)	7 (87.5)	88 (17)	0.02
Mean number of fragments (SD)	13.4 (3.3)	12.5 (4.4)	13.5 (3.4)	0.27
<12 biopsy fragments, n (%)	33 (12.3)	2 (14.3)	35 (12.4)	0.23
*Diabetes mellitus,* n (%)	84 (16.9)	1 (4.5)	16 (3.1)	0.09
Stroke, n (%)	15 (3)	1 (4.5)	16 (3.1)	0.68

LUTS: lower urinary tract symptoms; IPSS: International Prostate Symptoms Score.

**Figure 2:**
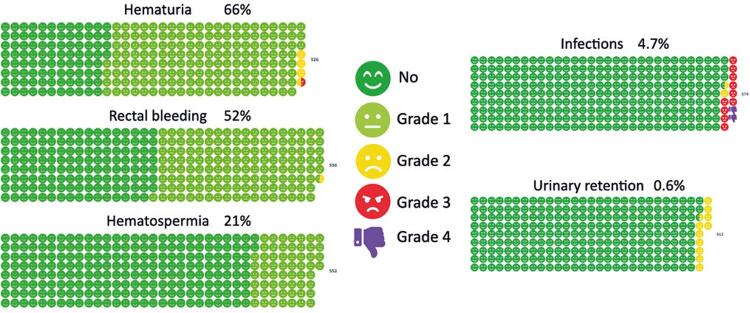
Complications classified according to the Clavien-Dindo classification

Overall, there were 20 hospitalizations and 37 visits to the emergency room or attending general physicians during the 28-day follow-up period (Table 5 and Figure 2). At the end of four weeks, the patients or their families returned to our referral center to retrieve the pathology results of the PBx. For patients referred from other institutions, the delivery of the anatomopathological study was conditional on the delivery of the self-completed form of complications that occurred during this period, as a mutual agreement. Although the complication form was the same paper that was used to deliver the results, 38% of the questionnaires were partially complete or left blank.

**Table 5:** Complications classified according to the Clavien-Dindo classification

Hemorragic complications	
Hematuria	344 (66)
Grade 1 mild without intervention	336 (64.6)
Grade 2 bladder irrigation	7 (1.3)
Grade 3 transfusion	1 (0.2)
Hematochezia	286 (52.1)
Grade 1 mild without intervention	285 (51.9)
Grade 2 medication	1 (0.2)
Hematospermia	116 (21)
Grade 1 observation	116 (21)
Infectious complications	27 (4.7)
Grade 1	-
Grade 2 antibiotics p.o.	3 (0.5)
Grade 3 antibiotic IV	20 (3.6)
Grade 4 sepsis; risk of death	4 (0.6)
Acute urinary retention	21 (4.1)
Grade 2 indwelling urinary catheter	21 (4.1)

IV: intravenous.

## DISCUSSION

This study included only patients from the Brazilian public healthcare system. Our study identified a significant association between younger age and an increased risk of hemorrhagic complications, presumably due to better vascular supply in this group and/or biopsy strategies obtaining more tissue samples in younger patients.^(7,8)^ Similarly, Kranse et al. found that younger patients and those with elevated PSA levels were more likely to experience such complications.^(9)^This population also tends to be more sexually active and consequently reports episodes of haematospermia during the 4-week follow-up period. Presumably, younger age, normal erectile function, and a higher rate of haematospermia coincide with men who are generally more active. This study also found that men with hypertension reported significantly more hemorrhagic events.

Infectious complications were observed in almost 5% of the patients, with significant associations found for urinary catheter use (p<0.001), a recent history of urinary tract infections (p<0.001), and recent quinolone use (p=0.001). These findings are consistent with the results of Tandoğdu et al., who noted that prior catheter use and antimicrobial resistance significantly contribute to post-biopsy infections.^(5)^ Similarly, Wagenlehner et al., in their Global Prevalence of Infections in Urology study, identified the use of quinolones as a contributing factor to the increasing rates of urosepsis in patients undergoing prostate biopsy.^(10)^ The high incidence of quinolone use in our cohort confirmed the need to reassess prophylactic antibiotic protocols, particularly in patients with recent antibiotic exposure. Nam et al. analyzed a Canadian database and observed an increase in the need for hospitalization after prostate biopsy from 1% to 4% in patients who underwent PBx over a 10-year period up to 2005.^(11)^ The rise in antibiotic resistance is particularly alarming. By 2050, six of the most dangerous pathogens are projected to surpass cancer as the leading cause of death. Among these so-called “superbugs”, *Escherichia coli*, *Klebsiella pneumoniae*, and *Pseudomonas aeruginosa* are frequently implicated in urinary tract infections and other deadly infections. In South America, the economic burden of antibiotic resistance is expected to reach 100 trillion USD annually.^(10, 12-14)^

Acute urinary retention was noted in 4.1% of patients. The following were associated with AUR: moderate to severe LUTS, a prostate volume greater than 89mL, a thickened bladder wall, and an IPP ≥10mm. These findings are consistent with those of a meta-analysis by Lim et al., who found that larger prostate volumes and the presence of a median lobe increased the risk of urinary obstruction following biopsy.^(15,16)^ Despite the lack of consensus, the use of non-invasive tests, such as ultrasound of the lower urinary tract for measuring post-void residual volume, the evaluation of IPP, and bladder wall thickness, or even findings from MRI screening, may highlight the presence of these anatomical features during pre-procedural assessment and encourage increased care during the peri-biopsy period.

Regardless of the recent international recommendations advocating for the preferential use of the transperineal approach due to its association with lower infection rates and reduced antibiotic usage, the dissemination of this practice within Brazil’s Public Healthcare System (SUS – *Sistema Único de Saúde*) remains limited to very few centers.^(4,17)^ This is primarily due to the high costs associated with acquiring ultrasound equipped with transperineal-specific probes, as well as outdated reimbursement policies. The current value for a prostate biopsy in SIGTAP (Brazilian Unified Health System Procedure Table) (USD 38.00) is insufficient to cover the costs of disposable materials, medications, and professional fees.

Loeb et al. reported a post-biopsy hospitalization rate of 6.9% in the SEER database, which correlated with non-white patients.^(18)^ In the present study, a lower hospitalization rate of 3.5% was observed, and no significant correlation was observed between ethnicity and post-biopsy complications.

Although this study included over 1,000 men, potentially representing the largest prospective prostate biopsy cohort in Brazil, it had several limitations. First, the loss of follow-up for 38% of the patients who did not return their properly completed questionnaires at the 28-day follow-up may have introduced a selection bias, potentially impacting the generalizability of the findings. Moreover, reliance on patient-reported data and clinical records presents challenges concerning the accuracy and completeness of the information collected, particularly in populations with low levels of education. As noted by Çek et al. in their multinational studies, variations in healthcare practices across centers can also affect complication rates, making it difficult to compare findings universally.^(19)^ Furthermore, the low educational level of the population may have influenced the patients’ understanding of the study and their adherence to follow-up, potentially leading to the underreporting of complications. The period covered by this series reflects the early stages of our local experience with MRI-guided targeted biopsies. Except for those under active surveillance who were followed up within the institution and had undergone prior MRI, only a minority of the referred patients had undergone further investigation with multiparametric MRI (14.7%). Nevertheless, although Prostate Imaging Reporting and Data System findings influence cancer detection rates and biopsy indications, the reality of public healthcare in many regions of the country is reflected in this cohort population. Finally, the study was conducted at a single center, with all procedures performed by the same urologist, which may not reflect the full spectrum of microbial resistance patterns or complication rates observed in other regions.

## CONCLUSION

Clinically significant (Clavien-Dindo Grades 3–5) adverse events are extremely rare after transrectal prostate biopsies. While our findings offer important insights into the risk factors associated with postbiopsy complications, further research is warranted to refine preventive strategies. Careful preprocedural evaluation of antimicrobial use, particularly quinolones, along with the assessment of identified risk factors, can help improve patient safety.
